# Structural and functional analysis of the human spliceosomal DEAD-box helicase Prp28

**DOI:** 10.1107/S1399004714006439

**Published:** 2014-05-24

**Authors:** Sina Möhlmann, Rebecca Mathew, Piotr Neumann, Andreas Schmitt, Reinhard Lührmann, Ralf Ficner

**Affiliations:** a Molecular Structural Biology, Georg-August-University Göttingen, Justus-von-Liebig Weg 11, 37077 Göttingen, Germany; b Cellular Biochemistry, Max-Planck-Institute for Biophysical Chemistry, Am Fassberg, 37077 Göttingen, Germany

**Keywords:** Prp28, DEAD-box proteins, helicase domain

## Abstract

The DEAD-box protein Prp28 is essential for pre-mRNA splicing as it plays a key role in the formation of an active spliceosome. Prp28 participates in the release of the U1 snRNP from the 5′-splice site during association of the U5·U4/U6 tri-snRNP, which is a crucial step in the transition from a pre-catalytic spliceosome to an activated spliceosome. Here, it is demonstrated that the purified helicase domain of human Prp28 (hPrp28ΔN) binds ADP, whereas binding of ATP and ATPase activity could not be detected. ATP binding could not be observed for purified full-length hPrp28 either, but within an assembled spliceosomal complex hPrp28 gains ATP-binding activity. In order to understand the structural basis for the ATP-binding deficiency of isolated hPrp28, the crystal structure of hPrp28ΔN was determined at 2.0 Å resolution. In the crystal the helicase domain adopts a wide-open conformation, as the two RecA-like domains are extraordinarily displaced from the productive ATPase conformation. Binding of ATP is hindered by a closed conformation of the P-loop, which occupies the space required for the γ-phosphate of ATP.

## Abstract 2

The crystal structure of the helicase domain of the human spliceosomal DEAD-box protein Prp28 was solved by SAD. The binding of ADP and ATP by Prp28 was studied biochemically and analysed with regard to the crystal structure.

## 1. Introduction   

Many eukaryotic genes contain noncoding introns that are excised from pre-mRNA prior to the transport of mature mRNA out of the nucleus into the cytoplasm. This pre-mRNA splicing process comprises two consecutive transesterification reactions catalyzed by the spliceosome, a multi-megadalton ribonucleoprotein (RNP) complex. For each intron to be removed the spliceosome forms anew by the ordered assembly of five small nuclear RNPs (U1, U2, U4, U5 and U6 snRNPs) and several additional proteins on the pre-mRNA intron (Will & Lührmann, 2011 ▶). Initially, the U1 and U2 snRNPs bind to the pre-mRNA at the 5′ splice site and the branch-point sequence (BPS), respectively, forming the spliceosomal A complex. The pre-assembled U4/U6·U5 tri-snRNP then joins, generating the B complex. Subsequently, the spliceosome is transformed into the catalytically active B* complex, which is capable of catalyzing the first of the two splicing reactions. After the first splicing reaction, the C complex is formed and the second transesterification reaction takes place. Finally, the spliced mRNA is displaced from the spliceosome and the intron-lariat spliceosome is disassembled.

During assembly, activation and disassembly the composition of the spliceosome changes substantially, *e.g.* the U1 and U4 snRNPs are released during the formation of an active spliceosome, and profound rearrangements of the RNA–RNA interaction network occur (Wahl *et al.*, 2009 ▶; Hoskins & Moore, 2012 ▶). In the A complex, U1 snRNA base pairs with the 5′  splice site and U2 snRNA with the BPS and, within the intact U4/U6·U5 tri-snRNP, the U4 and U6 snRNAs bind to each other by two double-stranded helices. After complex B formation U4 is displaced from U6, allowing U6 snRNA to form an intramolceular stem-loop (U6-ILS) and to engage in new base-pair formations with U2 snRNA. At the same time, the U6 ACAGA sequence interacts with the 5′ end of the intron. During the disassembly of the spliceosome the RNA–RNA interactions are entirely disrupted so that the snRNAs can engage in a new round of splicing. The various structural rearrangements of the spliceosome during a splicing event are mainly driven by eight different ATP-dependent DE*x*D/H-box proteins (Staley & Guthrie, 1998 ▶; Schwer, 2001 ▶; Cordin *et al.*, 2012 ▶; Cordin & Beggs, 2013 ▶). DE*x*D/H-box proteins belong to the superfamily II of helicases and are involved in many cellular processes (Linder & Jankowsky, 2011 ▶; Jankowsky & Fairman, 2007 ▶). They are thought to use the energy of ATP hydrolysis to unwind dsRNA helices and/or to rearrange RNA–protein complexes. This activity is assigned to the conserved helicase core comprising about 400 residues, which fold into two RecA-like domains. Characteristic of DE*x*D/H-box proteins are the eight conserved short sequence motifs I, Ia, Ib, II, III, IV, V and VI that mediate ATPase and ‘RNA helicase’ activities. Motif I contains the P-loop with the canonical sequence T/S-G-T/S-G-K-T. The conserved residues of the P-loop make contacts with the phosphate moiety of the nucleotide, where negative charges are neutralized by contacts with the side chain of the conserved lysine and by hydrogen bonds to amides of the P-loop main chain. Motif II has the characteristic consensus sequence DE*x*D/H and provided the name of this protein family. The subfamily of DEAD-box proteins shares three additional sequence motifs, denoted Q, GG and Q*xx*R. Based on mutagenesis and structural studies, motifs Q, I (also known as the Walker A motif), II (also known as the Walker B motif), V and VI are involved in ATP binding and hydrolysis. RNA binding is mediated by motifs Ia, Ib, IV and V. Motif III is assumed to be important for coupling ATP hydrolysis to the RNA-unwinding activity (Cordin *et al.*, 2006 ▶; Hilbert *et al.*, 2009 ▶).

Two RNA helicases are involved in the transitions from a pre-catalytic spliceosome to an activated spliceosome (A to B to B* complex), namely the DEAD-box protein Prp28 and the Ski2-like helicase Brr2. Both are essential and integral parts of the human U5 snRNP, whereas in yeast only Brr2 is tightly associated with the U5 snRNP. Brr2 has been shown to unwind the U4/U6 snRNA duplex, leading to release of the U4 snRNP (Laggerbauer *et al.*, 1998 ▶; Raghunathan & Guthrie, 1998 ▶), and genetic studies in yeast suggested that yeast Prp28 (yPrp28) is involved in the displacement of the U1 snRNP from the 5′ splice site (Staley & Guthrie, 1999 ▶; Chen *et al.*, 2001 ▶; Ismaili *et al.*, 2001 ▶). Additionally, yPrp28 has been found to perform a proof-reading function during this step of spliceosome assembly (Yang *et al.*, 2013 ▶). Interestingly, no ATPase or helicase activity could be detected for isolated Prp28 *in vitro* (Strauss & Guthrie, 1994 ▶; Laggerbauer *et al.*, 1998 ▶; Yang *et al.*, 2013 ▶); however, extensive mutagenesis studies concerning residues of the conserved motifs demonstrated a requirement for the Prp28 ATPase and helicase activities in pre-mRNA splicing (Chang *et al.*, 1997 ▶). Recently, a second and ATP-independent function of Prp28 during formation of the spliceosomal commitment complex 2 was found (Price *et al.*, 2014 ▶). Human Prp28 (hPrp28; also denoted U5-100K) comprises 820 amino-acid residues. The helicase core with the conserved sequence motifs corresponds to the C-terminal half of the protein. In contrast to yPrp28, hPrp28 contains about 230 additional N-terminal residues, which are rich in arginine/serine as well as arginine/glutamate and arginine/aspartate dipeptide motifs and therefore have been annotated the RS-like domain (Teigelkamp *et al.*, 1997 ▶). Phosphorylation of this RS-like domain by the kinase SRPK2 promotes a dynamic switch which is required for the association of hPrp28 with the U4/U6·U5 tri-snRNP and the formation of the spliceosomal B complex (Mathew *et al.*, 2008 ▶; Xiang *et al.*, 2013 ▶).

Here, we report the crystal structure of hPrp28ΔN, a fragment of human Prp28 comprising the entire helicase core and a short N-terminal extension (NTE). The structure is composed of two RecA-like domains, similar to other DEAD-box proteins. *In vitro*, hPrp28 shows neither ATPase activity nor ATP binding. These properties are consistent with the nonproductive arrangement of the RecA-like domains and the partially closed conformation of the ATP binding site observed in the crystal structure of hPrp28ΔN. Interacting proteins and/or RNA inside the spliceosome might abrogate the inhibition of the ATPase activity, ensuring the precise location and timing of the action of hPrp28.

## 2. Materials and methods   

### 2.1. Protein expression and purification   

Full-length hPrp28 was overexpressed, purified and phosphorylated as described previously (Mathew *et al.*, 2008 ▶). The cDNA fragment encoding hPrp28ΔN (residues 338–820) was cloned into the vector pGEX-6P3. The GST-fused recombinant protein was expressed in *Escherichia coli* BL21 Star (DE3) cells at 18°C. Cells were disrupted using a fluidizer (Microfluidics) in 50 m*M* Tris–HCl pH 7.5, 2 *M* LiCl, 5%(*v*/*v*) glycerol, 2 m*M* β-mercaptoethanol. The protein was purified at 4°C on a Glutathione Sepharose column [in 50 m*M* Tris–HCl pH 7.5, 500 m*M* NaCl, 5%(*v*/*v*) glycerol, 2 m*M* β-mercaptoethanol], followed by cleavage of the GST tag with PreScission protease. Further purification was obtained using a Superdex 75 gel-filtration column and residual GST was subsequently removed using a Glutathione Sepharose column. The protein was concentrated to 15 mg ml^−1^ in 10 m*M* Tris–HCl pH 7.5, 150 m*M* NaCl, 2 m*M* β-mercaptoethanol.

### 2.2. Determination of nucleotide-binding constants   

The equilibrium dissociation constants for ADP and ATP were determined by fluorescence titration using mant-labelled nucleotides. Each reaction consisted of 4 µ*M* hPrp28ΔN protein, mant-ADP or mant-ATP in the range between 1 and 400 µ*M*, 10 m*M* HEPES–NaOH pH 7.5, 150 m*M* NaCl, 10 m*M* MgCl_2_ and 1 m*M* DTT. Prior to the measurements, the samples were incubated for 45 min at 25°C in order to reach an equilibrium state. Fluorescence was measured using a Fluoromax-3 spectrofluorimeter (Horiba Jobin Yvon) with an excitation wavelength of 295 nm and recording the emission of tryptophans as counts per second (cps) at 330 nm with an integration time of 0.5 s. Measurements were performed independently three times for every sample. The measured values were corrected for the inner filter effect (Birdsall *et al.*, 1983 ▶) and *K*_d_ was calculated by nonlinear regression to the Michaelis–Menten equation using *SigmaPlot*.

### 2.3. Determination of ATP-hydrolysis rates   

In order to analyze the ATPase activity of the purified hPrp28, a HPLC-based activity assay was applied. Purified hPrp28 at a concentration of 1 mg ml^−1^ in a buffer consisting of 20 m*M* Tris–HCl pH 7.5, 300 m*M* NaCl, 5 m*M* MgCl_2_, 1 m*M* DTT was incubated with 0.5 m*M* ATP in the presence or absence of 5 µg µl^−1^ yeast RNA type III at 37°C for 9 h. A reaction mixture without hPrp28 was prepared as a control. Samples were taken at distinct time points and the reaction was stopped by incubation at 85°C for 2 min. Precipitated protein was pelleted by centrifugation (14 000*g*, 5 min, 4°C) and the supernatant was loaded onto a reversed-phase HPLC column (Prontosil C18-AQ, Bischoff Chromatography, Germany), which was equilibrated in a buffer consisting of 100 m*M* K_2_HPO_4_/KH_2_PO_4_ pH 6.5. The elution of ATP and ADP was monitored by UV absorption at 254 nm, with commercially available ATP and ADP serving as references for column calibration. The ATP and ADP peak areas were integrated and values for the sample in the absence of protein were substracted to correct for background hydrolysis of ATP. The amount of hydrolyzed ATP was determined using the following equation: hydrolyzed ATP as a percentage = 100% × area_ADP_/(area_ATP_ + area_ADP_).

### 2.4. ATP-hPrp28 UV cross-linking   

Purified His-tagged hPrp28 (30 pmol) in a total volume of 50 µl was incubated under splicing conditions (60% nuclear extract, 20 m*M* creatine phosphate, 3 m*M* MgCl_2_, 10 n*M* of MINX pre-mRNA with 30 µCi α^32^P ATP; specific activity 3000 Ci mmol^−1^) for 15 min at 30°C. After incubation, the samples were transferred onto ice and cross-linked using a Sylvania G8T5 germicidal UV lamp for 5 min at a distance of 2 cm. The cross-linked proteins were then immunoprecipitated with an anti-His antibody (Qiagen). For the immunoprecipitations, 4 µg anti-His antibody was coupled to 20 µl protein A Sepharose. The cross-linked samples together with 200 µl IPP_500_ buffer (20 m*M* Tris–HCl pH 8.0, 500 m*M* NaCl, 0.1% Triton X-100) were added to the washed beads, and hPrp28 was precipitated from the reactions for 2 h at 4°C. The beads were subsequently washed three times with 1 ml IPP_500_ and precipitated proteins were extracted by boiling the beads in protein loading buffer. After extraction, the proteins were separated by 10% SDS–PAGE. The gel was silver-stained and cross-linked proteins were detected by autoradiography. In the absence of nuclear extract, 30 pmol purified protein (50 µl total reaction volume) was incubated in 75 m*M* KCl, 50 m*M* Tris–HCl pH 8.0, 1.5 m*M* MgCl_2_, 1.25 m*M* DTT in the presence or absence of 0.6 µg µl^−1^ poly-U.

### 2.5. Crystallization and derivatization   

Crystals were grown at 20°C by sitting-drop vapour diffusion in a condition consisting of 2.2 *M* ammonium sulfate, 20%(*v*/*v*) glycerol, 0.1 *M* CAPS pH 9.0 by mixing 1 µl protein solution with 1 µl reservoir solution. For crystal soaking, 5 m*M* phenylmercuryacetate was dissolved in the same buffer and the crystals were incubated for 7 d at 20°C.

### 2.6. Data collection and processing   

Anomalous X-ray diffraction data were obtained at a temperature of 100 K on beamline 14.1 at HZB/BESSY II (Mueller *et al.*, 2012 ▶) equipped with a MAR Mosaic 225 mm CCD. Diffraction data from a single mercury-derivative crystal were collected at two wavelengths: peak (1.00858 Å) and low-energy remote (1.01212 Å) with regard to the Hg edge. Oscillation photographs were integrated using *MOSFLM* (Battye *et al.*, 2011 ▶) for the low-energy remote data and *XDS* (Kabsch, 2010*a* ▶) for the peak data. Diffraction data were merged and scaled with *SCALA* from the *CCP*4 suite (Winn *et al.*, 2011 ▶) and *XSCALE* (Kabsch, 2010*b* ▶), respectively.

### 2.7. Structure solution and refinement   

SAD phasing using *SHARP*/*autoSHARP* (Vonrhein *et al.*, 2007 ▶) employing *SHELXD* (Schneider & Sheldrick, 2002 ▶) for heavy-atom substructure location and *SOLOMON* (Abrahams & Leslie, 1996 ▶) for density modification produced interpretable maps with a clear protein–solvent boundary. A single heavy-atom site at residue Cys543 showed positional disorder with refined occupancies of 0.47 and 0.33 (Supplementary Fig. S1^1^). Model building was performed by fitting fragments of the known structure of the DEAD-box protein Vasa (PDB entry 2db3; Sengoku *et al.*, 2006 ▶) followed by verification against simulated-annealing (SA) OMIT maps using *Coot* (Emsley *et al.*, 2010 ▶). The initial model was refined against the 2.0 Å resolution low-energy remote data in *CNS* (Brunger, 2007 ▶) employing slow-cooling SA dynamics. To model anisotropic displacements of the domains, the final refinement steps were performed in *PHENIX* (Adams *et al.*, 2010 ▶) with four TLS groups and resulted in a crystallographic *R* and *R*_free_ value of 0.193 and 0.218, respectively. The final hPrp28ΔN model consists of residues 352–806, five sulfate ions, two glycerol molecules, 3-cyclohexyl-1-propylsulfonic acid, one disordered Hg atom and 220 solvent molecules. Missing residues, namely the N-terminal residues 338–351, loops 695–702 and 724–727 and the C-terminal residues 807–820, resulted from non-interpretable electron density and are most likely to be disordered. The refined model has good geometry as judged by *RAMPAGE* (98.7% of residues in the favoured region, 1.4% in the allowed region). A search for similar structures was performed with *DALI* (Holm *et al.*, 2008 ▶). The atomic coordinates of the hPrp28ΔN structure have been deposited in the Protein Data Bank (PDB entry 4nho).

## 3. Results and discussion   

### 3.1. ADP/ATP-binding properties of hPrp28ΔN   

**Figure 1:**
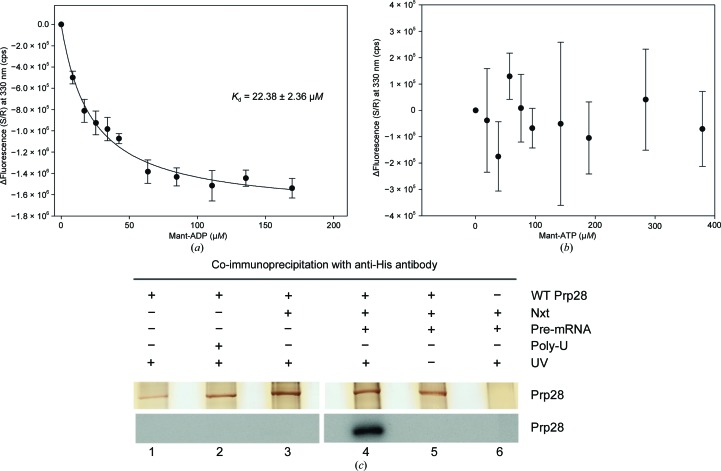
Adenosine nucleotide binding of hPrp28 determined by (*a*, *b*) fluorescence titration using mant-ADP or mant-ATP and (*c*) UV-induced cross-linking using α-^32^P-labelled ATP. (*a*, *b*) The difference in corrected fluorescence signal is plotted against the ADP/ATP concentration and fitted to the Michaelis–Menten equation. For mant-ADP, a *K*_d_ of 22.4 ± 2.4 µ*M* was calculated, while in the case of mant-ATP curve fitting was not possible. (*c*) UV-induced cross-linking of α-^32^P ATP to purified hPrp28. 30 pmol purified hPrp28 was incubated in the absence of nuclear extract (Nxt; lanes 1–2) without (lane 1) or with (lane 2) poly-U (0.6 µg µl^−1^) or in the presence of nuclear extract (lanes 3–6) in the absence (lane 3) or presence (lane 4) of MINX pre-mRNA under splicing conditions (see §[Sec sec2]2) and subjected to UV irradiation. After cross-linking, hPrp28 was immunoprecipitated with anti-His antibody. The precipitates were loaded onto a 10% SDS–PAGE and radioactive hPrp28 (cross-linked to ATP) was detected by autoradiography. Cross-linking of ATP to hPrp28 was also assayed in the absence of UV irradiation (lane 5). As control for immunoprecipitation the reaction was carried out without purified hPrp28 (lane 6).

Full-length hPrp28 and an N-terminally truncated hPrp28 variant (hPrp28ΔN) which consists of the entire helicase domain were cloned, overexpressed and purified. Both full-length hPrp28 containing the phosphorylated RS-domain and hPrp28ΔN did not show significant ATPase activity, with a turnover of 8.3 × 10^−5^ s^−1^ in the case of hPrp28ΔN, which could not be stimulated in the presence of RNA. This raises the question of whether ATP cannot be bound by hPrp28 or whether ATP is bound but not hydrolyzed by hPrp28. Therefore, we analyzed the binding of ADP and ATP to hPrp28ΔN by means of fluorescence spectroscopy using mant-ADP and mant-ATP, respectively (Figs. 1 ▶ *a* and 1 ▶ *b*). ADP binds to hPrp28ΔN with a dissociation constant (*K*_d_) of 22.4 ± 2.4 µ*M*, which is comparable to that of other DEAD-box proteins. No interaction with ATP was observed for ATP concentrations up to 400 µ*M*, indicating a *K*_d_ for ATP of ≫400 µ*M*. This value is in good agreement with the *K*_d_ values of ATP for other DEAD-box proteins, which typically range between 70 µ*M* and >6 m*M* (Lorsch & Herschlag, 1998 ▶; Cao *et al.*, 2011 ▶; Henn *et al.*, 2008 ▶; Karow *et al.*, 2007 ▶; Hilbert *et al.*, 2011 ▶). Owing to limitations of the fluorescence-based nucleotide-binding assay caused by the inner filter effect, ATP concentrations above 400 µ*M* were not feasible.

### 3.2. hPrp28 binds ATP in the spliceosome   

Since previous mutagenesis studies on conserved sequence motifs of yPrp28 demonstrated that the ATPase activity of Prp28 is necessary for the splicing reaction (Chang *et al.*, 1997 ▶; Staley & Guthrie, 1999 ▶), we analyzed the binding of ATP to full-length hPRp28 under *in vitro* splicing conditions by means of UV-induced cross-linking using α^32^P-labelled ATP. Neither isolated hPrp28 nor hPrp28 in the presence of nuclear extract, which contains all of the spliceosomal proteins and snRNPs, could be cross-linked to ATP. However, in the presence of both nuclear extract and a pre-mRNA, *i.e.* under conditions of spliceosome formation, ATP was cross-linked to hPrp28 (Fig. 1 ▶ *c*). This indicates that Prp28 can bind ATP only when it is part of a spliceosomal complex assembled on an intron-containing pre-mRNA. Since α^32^P-labelled ATP was used in this experiment, cross-linked ADP originating from ATP hydrolysis would also be detected. However, in the absence of pre-mRNA no nucleotide was cross-linked to hPrp28, indicating that the signal in the presence of pre-mRNA indeed results from bound ATP.

### 3.3. Structure determination of hPrp28ΔN   

In order to understand the structural basis for the lack of ATP-binding activity of hPrp28 in its free form, we set out to determine the crystal structure of hPrp28. All attempts to crystallize full-length hPrp28 have failed, which is probably owing to the N-terminal RS-like domain, which was predicted to be intrinsically disordered (Korneta *et al.*, 2012 ▶). Well diffracting crystals of hPrp28ΔN could be obtained and the crystal structure was solved by means of SAD using a heavy-atom derivative. The structure was refined at a resolution of 2.0 Å and contains residues 352–806. X-ray data-collection and crystal structure-refinement statistics are summarized in Tables 1 ▶ and 2 ▶. Two loop regions (residues 695–702 and 724–727) as well as the N-terminal residues 338–351 and the C-terminal residues 807–820 are not defined in the electron-density map and are most likely to be disordered.

### 3.4. Overall structure   

**Figure 2:**
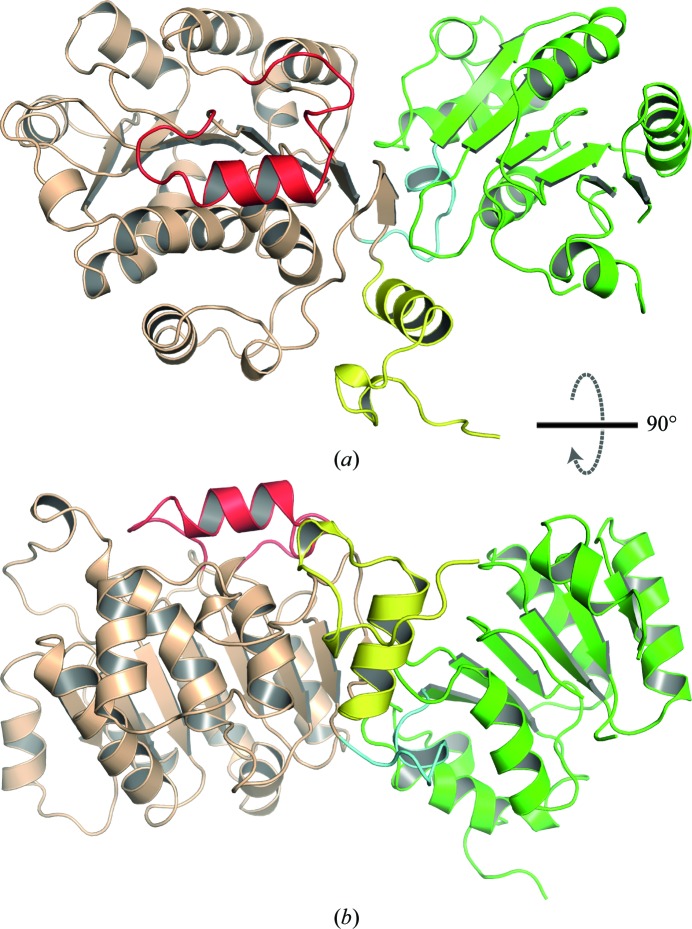
Two perpendicular views (*a*, *b*) of the hPrp28ΔN structure. The N-­terminal extension is coloured yellow, the RecA-1 domain brown and the RecA-2 domain green. Additionally, the insertion in RecA-1 is highlighted in red and the linker connecting RecA-1 and RecA-2 is coloured light blue.

The three-dimensional structure of hPrp28ΔN can be divided into four parts: an N-terminal extension (NTE; residues 352–378), the N-terminal RecA-like domain (RecA-1; residues 379–629), the linker region (residues 630–639) and the C-terminal RecA-like domain (RecA-2; residues 640–806) (Fig. 2 ▶, Supplementary Fig. S2). The NTE belongs to a region (residues 221–378) that connects the helicase domain to the N-terminal RS-like domain (residues 1–220), which is present only in Prp28 from higher eukaryotes. Residues 367–377 of the NTE form an α-helix which packs against RecA-1 (Supplementary Fig. S3). Interestingly, this helix is also found in an identical position in the DEAD-box protein DDX5 (PDB entries 4a4d and 3fe2; Dutta *et al.*, 2012 ▶; Schutz *et al.*, 2010 ▶). The further N-terminal residues 353–366 form an irregular loop including two short helical turns; however, its position and conformation appear to be induced by interactions with a neighbouring protein molecule in the crystal lattice.

The fold of the two RecA-like domains is very similar to the known structures of other superfamily II (SF2) ATPases. The N-terminal RecA-like domain (RecA-1; residues 379–629) contains an eight-stranded β-sheet comprising seven parallel strands and one antiparallel strand, namely the first strand. A total of ten α-helices pack against this sheet on both sides. The fold of the hPrp28 RecA-1 domain is most similar to that of DDX5 (PDB entry 3fe2), with an r.m.s.d. of 1.7 Å for 231 common C^α^ atoms. A unique feature of hPrp28 is the insertion of 24 amino acids (residues 576–599), which form a protuberance on the RecA-1 domain (Supplementary Fig. S3). This insertion consists of an α-helix within an extended loop and is located on the opposite side with respect to the conserved sequence motifs of the DEAD-box protein family (Fig. 2 ▶). The insertion is also found in the sequence of Prp28 orthologues from metazoans, but is missing in Prp28 from *Saccharomyces cerevisiae*. The C-terminally located RecA-2 domain (residues 636–802) is smaller than the RecA-1 domain. It contains a six-stranded parallel β-sheet surrounded by five α-helices. The fold of the hPrp28 RecA-2 domain is most similar to that of DDX3 (Högbom *et al.*, 2007 ▶; PDB entry 2jgn) as the r.m.s.d. for 153 common C^α^ atoms amounts to 1.6 Å.

### 3.5. Helicase core conformation   

**Figure 3:**
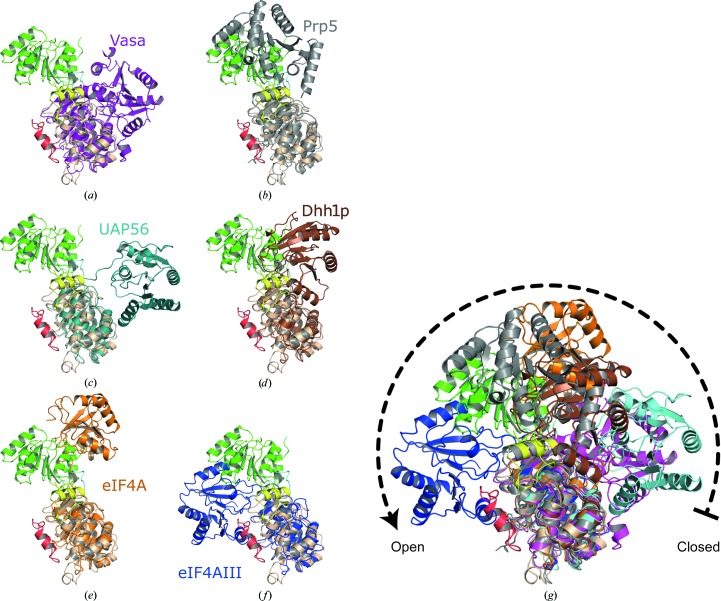
The helicase core of hPrp28ΔN has an open overall conformation as the two RecA domains are displaced with respect to the active conformation represented by Vasa (purple). Superposition of hPrp28ΔN with (*a*) Vasa in its active closed conformation and other DEAD-box proteins exhibiting an open conformation: (*b*) Prp5, (*c*) UAP56, (*d*) Dhh1p, (*e*) eIF4A and (*f*) eIF4AIII. (*g*) Superposition of (*a*)–(*f*).

**Figure 4:**
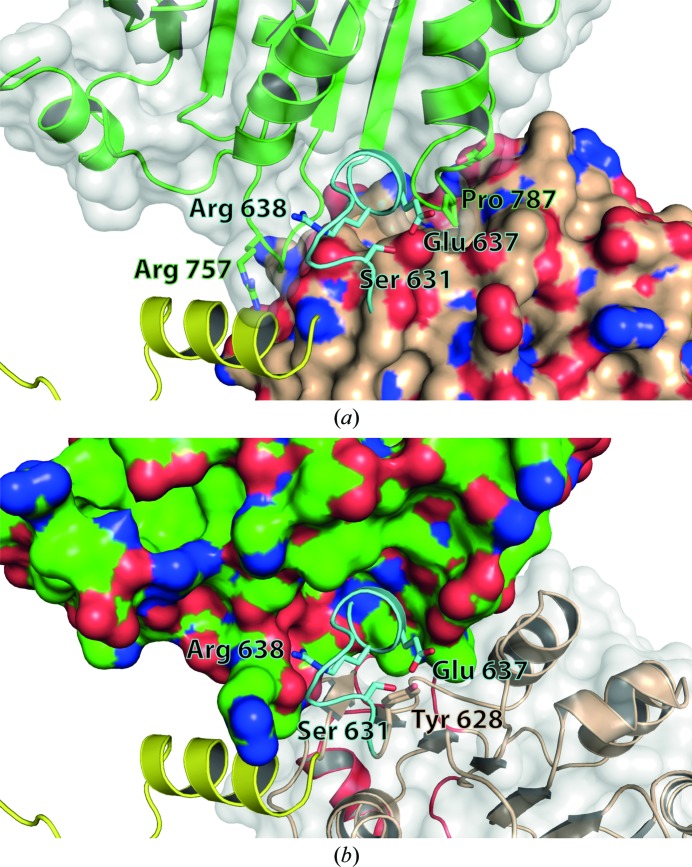
Interactions of the back side of the RecA-2 domain (green ribbon model) with the RecA-1 domain, the linker between the two RecA domains (blue) and the N-terminal extension (yellow). (*a*) The surface of RecA-1 is coloured gold and surface-exposed N and O atoms are coloured blue and red, respectively. (*b*) The surface of RecA-2 is coloured green and surface-exposed N and O atoms are coloured blue and red, respectively.

The crystal structures of several other DEAD-box proteins revealed a high variability in the relative orientation of their two RecA-like domains as a short and flexible linker connects them (Hilbert *et al.*, 2009 ▶). Hence, the different conformations found in crystals might primarily be stabilized by crystal-packing contacts, while in solution the helicase domains are flexible. In the active state DEAD-box helicases have to adopt a closed conformation, as was observed in the structure of Vasa helicase with bound RNA and a nonhydrolysable ATP analogue (Sengoku *et al.*, 2006 ▶). In this conformation the conserved sequence motifs of the DEAD-box protein family are in close proximity to each other and form the complete active site located in the cleft between the two RecA-like domains. In the crystal structure of hPrp28ΔN, the orientation of the two RecA-like domains is unique compared with the crystal structures of other DEAD-box proteins (Fig. 3 ▶). The arrangement is more open than in other DEAD-box proteins, with exception of eIF4AIII in its free form, which is the most open form. Interestingly, the defined arrangement of the hPrp28ΔN RecA domains appears to be a favourable one as there are many intramolecular contacts between the two RecA domains, *e.g.* a salt bridge between Lys382 and Glu640 and five hydrogen bonds (Ile379–Gly759, Thr381–Gly759, Thr381–Ser761, Lys382–Val788 and Glu614–Ser790). Furthermore, the linker between the two RecA domains seems to be arrested in its conformation owing to tight hydrogen bonds of Ser631 to Tyr628 and Glu637 (Fig. 4 ▶).

### 3.6. The P-loop of hPrp28ΔN adopts a unique ‘half-open’ conformation   

**Figure 5:**
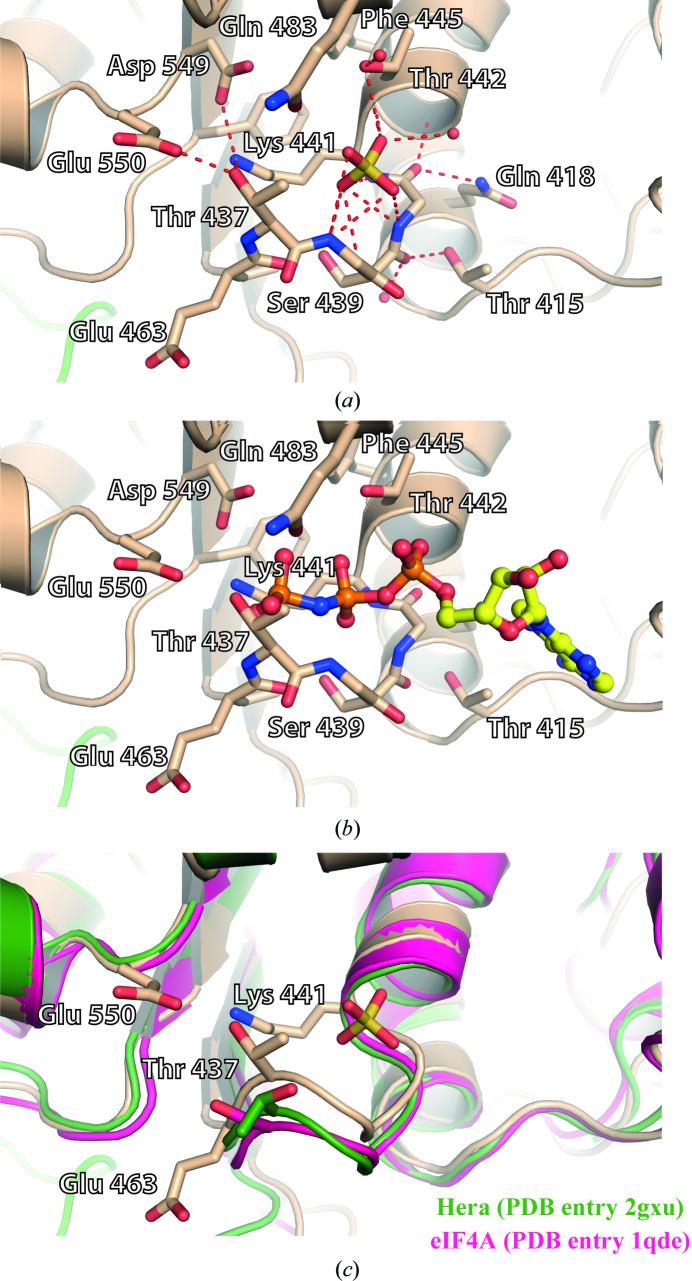
Conformation of the P-loop. (*a*) The conformation of the P-loop is stabilized by multiple hydrogen bonds (red dashed lines). The ATP binding pocket is occupied by a sulfate ion. (*b*) Model of bound ATP based on the Vasa–ATP complex structure. ATP binding is hindered by the P-loop owing to the hydrogen bonds between Thr437, Glu550 and Lys441. (*c*) The P-loop conformation of hPrp28ΔN is unique, as in the structures of eIF4A with bound sulfate or of the DEAD-box helicase Hera with bound phosphate the hydrogen bond between the Glu of the DEAD motif and the Thr or Ser of the P-loop is broken.

The ATP binding pocket in the RecA-1 domain is mainly composed of amino acids belonging to the sequence motifs Q, I and II. No nucleotide is bound to hPrp28ΔN, but its active site contains a sulfate ion (Fig. 5 ▶ *a*). Its binding site is almost identical to that of the sulfate and phosphate ions found in the DEAD-box proteins eIF4A (PDB entry 1qde) and Hera (PDB entry 2gxu), respectively (Benz *et al.*, 1999 ▶; Rudolph *et al.*, 2006 ▶). Based on a superposition with the crystal structure of Vasa (PDB entry 2db3; Sengoku *et al.*, 2006 ▶), an ATP molecule was modelled into the binding pocket of hPrp28ΔN (Fig. 4 ▶ *b*). The adenosine moiety fits well into the pocket and the α- and β-phosphate can also be accommodated. However, the γ-phosphate clashes with Thr437 of the P-loop, which corresponds to the conserved motif I (Fig. 5 ▶ *b*). The overall conformation of the P-loop is similar to that observed for other DEAD-box proteins with bound sulfate, phosphate or AMP, and was previously denoted as a half-open conformation (Supplementary Fig. S4). However, the position of Thr437 in the P-loop of hPrp28ΔN is shifted by about 2 Å towards the active site with respect to the common half-open conformation (Fig. 5 ▶ *c*). This unique P-loop conformation of hPrp28ΔN is stabilized by hydrogen bonds between Thr437, Glu550 and Lys441. These interactions tightly connect motifs I and II. Moreover, the conformation of the P-loop is affected by the bound sulfate ion located between the proposed binding sites of the α- and β-phosphates of the ATP. It is coordinated by hydrogen bonds to the amide N atoms of residues Gly438–Thr442 and the hydroxyl group of Thr442. A similar binding mode for a sulfate ion was observed in one of the structures of yeast eIF4A (Benz *et al.*, 1999 ▶). However, eIF4A in its free form is capable of binding ATP, which requires an opening of the binding site by moving the P-loop outwards, thereby allowing γ-phosphate binding. The superposition of eIF4A and hPrp28ΔN reveals that the conformation of their half-open P-loops is significantly different, which affects the length of the hydrogen bonds between Thr437, Glu550 and Lys441. While the distance of 2.5 Å between the hydroxyl group of Thr and the carboxylate group of Glu in the hPrp28ΔN structure indicates strong hydrogen bonding, the corresponding hydrogen bond is much longer in eIF4A, namely 3.5 Å in the eIF4A–sulfate complex structure (PDB entry 1qde) and 5.6 Å in the Hera–phosphate complex structure (PDB entry 2gxu). Additionally, in hPrp28ΔN Lys441 is within hydrogen-bonding distance (3.1 Å) of Thr437, while it is too distant in eIF4A (4.0 Å in PDB entry 1qde and 6.1 Å in PDB entry 2gxu). Hence, the much weaker interaction between motifs I and II in eIF4A as well as in Hera apparently facilitate opening of the P-loop upon ATP binding.

The P-loop of hPrp28ΔN is not involved in crystal contacts that could affect its conformation; hence, the structural determinant of its fixed conformation remains unclear. The P-loop and the surrounding residues are highly conserved among DEAD-box proteins. The only obvious difference in the P-loop of Prp28ΔN is found in the residue preceding the canonical sequence T/S-G-T/S-G-K-T, which is Gln or Lys in most DEAD-box proteins but is Glu (Glu434) in hPrp28. However, the side chain of Glu434 is not involved in any contacts with other residues and hence does not directly affect the position of Thr437.

### 3.7. Activation of Prp28   

Since the ATPase activity of Prp28 is essential for its function in the spliceosome, conformational changes in Prp28 have to occur in order for it to become prone to ATP binding and hydrolysis. Activation of Prp28 not only concerns the opening of the P-loop but also the rearrangement of the two RecA domains. The different relative positions of the RecA domains found in crystal structures of other DEAD-box proteins are thought to be a result of crystal packing, as the two RecA domains do not interact with each other in the absence of nucleotide or RNA ligand (Hilbert *et al.*, 2009 ▶). The linker connecting the RecA domains is believed to be flexible; however, its amino-acid sequence affects the degree of flexibility and consequently the activity of the helicase. This was demonstrated by the stepwise exchange of residues in the linker of eIF4A with the corresponding residues of Vasa, which significantly increased the ATPase activity of eIF4A (Low *et al.*, 2007 ▶). Interestingly, the linker of Prp28 appears to be arrested in its conformation by a tight network of hydrogen bonds, which consequently keeps the RecA domains in a wide-open state. Additionally, owing to the wide opening the RecA domains form many contacts with each other on their back sides, leading to a further stabilization of the wide-open state. The buried surface owing to interaction between the RecA-2 domain and the other parts of the protein molecule (including NTE, RecA-1 and the linker) is approximately 3496 Å^2^ (calculated with *PISA*; Krissinel & Henrick, 2007 ▶) and contains a hydrophobic core as well as polar and ionic interactions. These findings suggest that the wide-open state of Prp28 in the crystalline state could also be stable in solution and thus might explain the missing ATPase activity even in the presence of RNA. Since yPrp28 has a second and an ATP-independent function in the formation of the spliceosomal commitment complex 2 (Price *et al.*, 2014 ▶), it is intriguing to speculate whether Prp28 in its wide-open conformation participates in this early step of spliceosome assembly. This is strongly supported by analogy to the spliceosomal DEAD-box helicase Prp5, for which the open conformation observed in the crystal appears to be stabilized and importantly is essential for spliceosome assembly (Zhang *et al.*, 2013 ▶). The overall open conformations of Prp5 and Prp28, however, differ greatly (Fig. 3 ▶ *b*).

For the ATP-dependent function of Prp28 during formation of the spliceosomal B^act^ complex, conformational changes have to be induced in Prp28 by other components of the spliceosome. For some other DEAD-box proteins the structural basis for stimulation of ATPase and helicase activities by interacting proteins, such as eIF4G for eIF4A (Hilbert *et al.*, 2011 ▶) or Gle1 for Dbp5 (Montpetit *et al.*, 2011 ▶), is well known. In contrast, protein-interaction partners that form a stable complex with Prp28 and stimulate its activity are as yet unknown, but genetic screens in *S. cerevisiae* identified interactions with several proteins of the U1, U2, U5 and U6 snRNPs (Cordin & Beggs, 2013 ▶). Since previous ATP cross-linking studies suggest that hPrp28 within the U5 snRNP or U4/U6·U5 tri-snRNP does not bind ATP (Laggerbauer *et al.*, 1996 ▶), the interactions of hPrp28 with U5 snRNP or tri-snRNP proteins and snRNAs are not sufficient for activation. This is consistent with our finding that even the presence of all snRNPs in a nuclear extract does not induce ATP binding of hPrp28 and that the activation of hPrp28 takes place only after the tri-snRNP has joined the spliceosomal A complex. Hence, only in the framework of the spliceosome are the RecA domains of hPRp28 enforced to form the catalytically active conformation by as yet unknown interactions with spliceosomal proteins and/or snRNAs and pre-mRNA.

## Supplementary Material

Supplementary Figures.. DOI: 10.1107/S1399004714006439/dw5092sup1.pdf

PDB reference: Prp28, 4nho

## Figures and Tables

**Table 1:** Data collection and processing Values in parentheses are for the outer shell.

Data set	Low-energy remote	Peak
Wavelength (Å)	1.01212	1.00858
Temperature (K)	100	100
Space group	*C*222_1_	*C*222_1_
Unit-cell parameters		
*a* (Å)	125.43	125.36
*b* (Å)	136.77	136.65
*c* (Å)	73.22	73.15
α = β = γ (°)	90	90
Resolution range (Å)	39.97–2.00 (2.11–2.00)	36.00–2.00 (2.10–2.00)
No. of unique reflections	42906 (6219)	81386 (11104)
Completeness (%)	100.0 (100.0)	99.2 (96.2)
Multiplicity	4.9 (4.9)	2.6 (2.5)
〈*I*/σ(*I*)〉	12.1 (2.3)	15.42 (1.88)
*R*_merge_ (%)	6.4 (58.4)	3.8 (49.8)
CC_1/2_ † (%)	99.9 (95.2)	99.9 (99.2)
Anomalous correlation‡ (%)		29 (11)
Mean anomalous difference (SigAno)‡		1.12 (0.79)

† Calculated with *XSCALE* or *SCALA*; CC_1/2_ is the correlation coefficient between two randomly selected half-data sets as described by Karplus & Diederichs (2012 ▶).

‡ Calculated with *XSCALE*.

**Table 2:** Structure solution and refinement Values in parentheses are for the outer shell.

Resolution range (Å)	28.8250–2.0000 (2.0465–2.0000)
Completeness (%)	99.9
σ Cutoff	*F* > 1.340σ(*F*)
No. of reflections, working set	42839 (2699)
No. of reflections, test set	2159 (123)
Final *R*_cryst_	0.193 (0.3380)
Final *R*_free_	0.218 (0.3970)
No. of non-H atoms	
Protein	3546
Ligand	67
Water	220
R.m.s. deviations	
Bonds (Å)	0.012
Angles (°)	1.276
Average *B* factors (Å^2^)	
Protein	59.0
Ligand	67.3
Water	56.8
Ramachandran plot	
Favoured regions (%)	98.65
Outliers (%)	0.23

## Footnotes

1 Supporting information has been deposited in the IUCr electronic archive (Reference: DW5092).
