# Supplementary material for: Structural and functional analysis of the human spliceosomal DEAD-box helicase Prp28
Source: Acta Crystallogr D Biol Crystallogr. 2014 May 24;70(Pt 6):1622–30. doi: 10.1107/S1399004714006439 (PMC4051504; doi:10.1107/S1399004714006439)
Supplement: Supplementary file 1 [file d-70-01622-sup1.pdf]

Acta Crystallographica Section D

Volume 70 (2014)

Supporting information for article:

Structural and functional analysis of the human spliceosomal DEAD-box helicase Prp28

Sina Möhlmann, Rebecca Mathew, Piotr Neumann, Andreas Schmitt, Reinhard Lührmann and Ralf Ficner

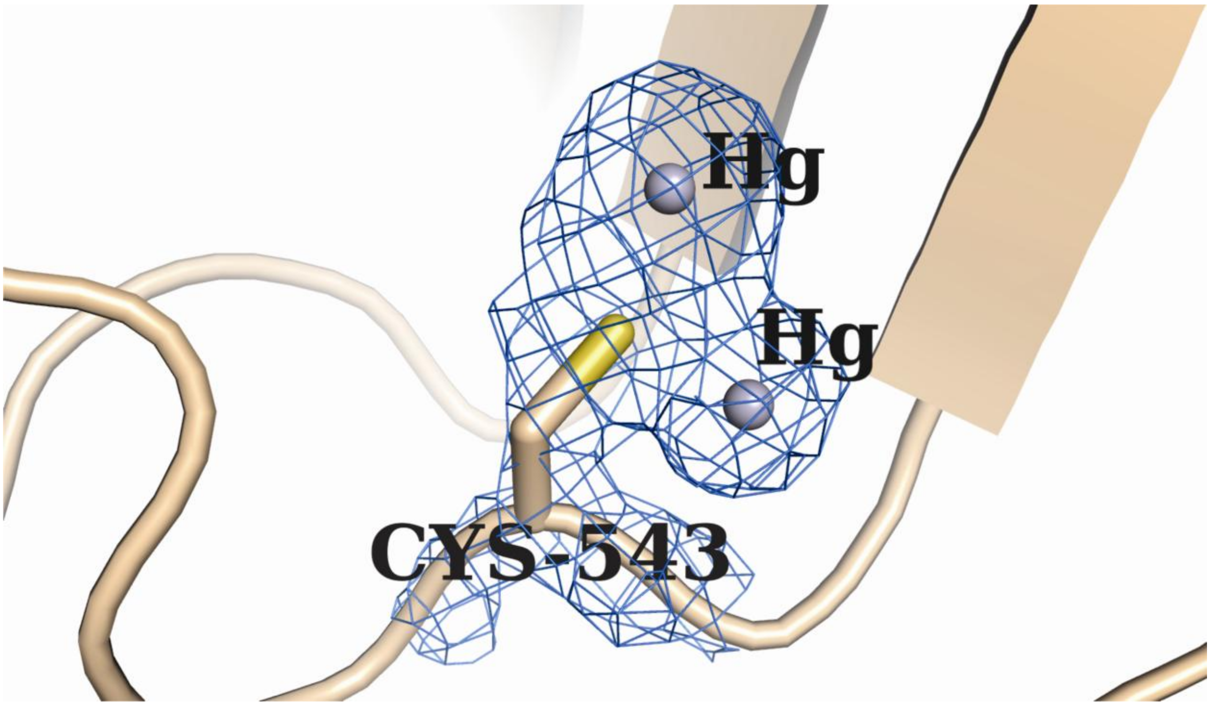

Figure S1 Fragment of the final model of hPrp28ΔN structure. Closer view on cysteine 543 shown as sticks and disordered Hg atom depicted as spheres together with final 2mFo-DFc electron density map contoured at 2 sigma level.

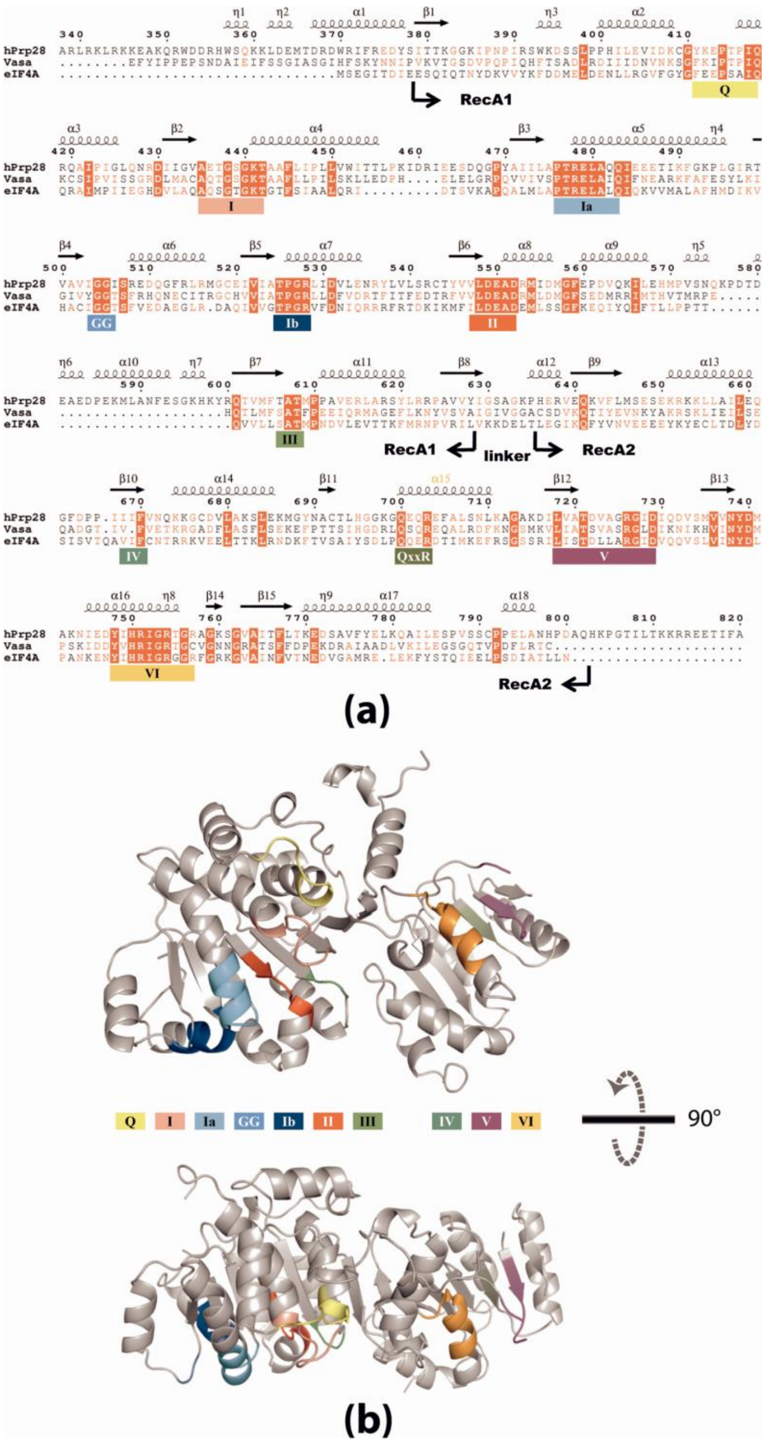

Figure S2 (a) Structure-based sequence alignment of the DEAD-box proteins hPrp28, Vasa, and eIF4A. The conserved sequence motifs (Q, I – VII) are highlighted, the conserved P-loop is part of motif I. The assignment of secondary structure elements is based on the hPrp28 crystal structure. The borders of the hPrp28 domains (NTE, RecA1, RecA2) are indicated. (b) Location of the conserved sequence motifs in the 3D structure of hPrp28.

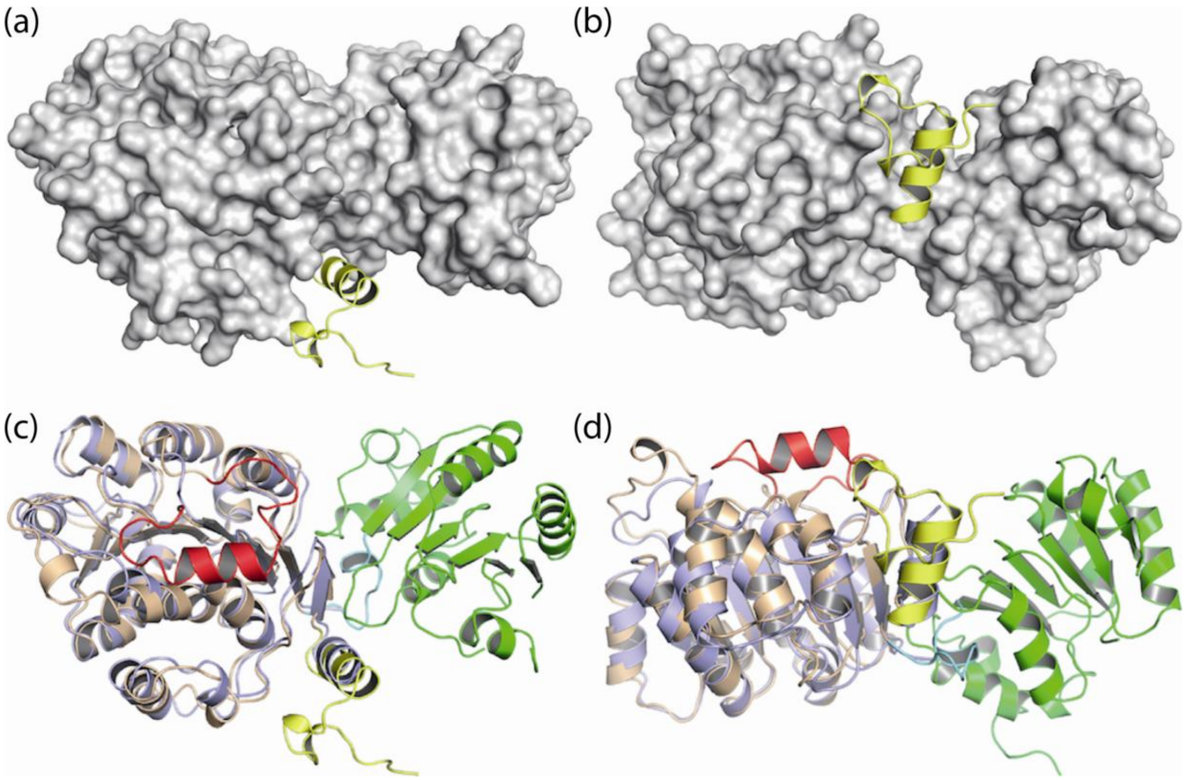

Figure S3 The N-terminal extension (NTE) of hPrp28ΔN (A,B) Two perpendicular views of hPrp28ΔN showing the RecA domains as surface and the NTE in a Ribbon representation. The NTE helix and the N-terminal tail mainly interact with RecA-1 domain. (C, D) A similar NTE helix is found in DDX5 (colored violet) shown in superposition with hPrp28ΔN.

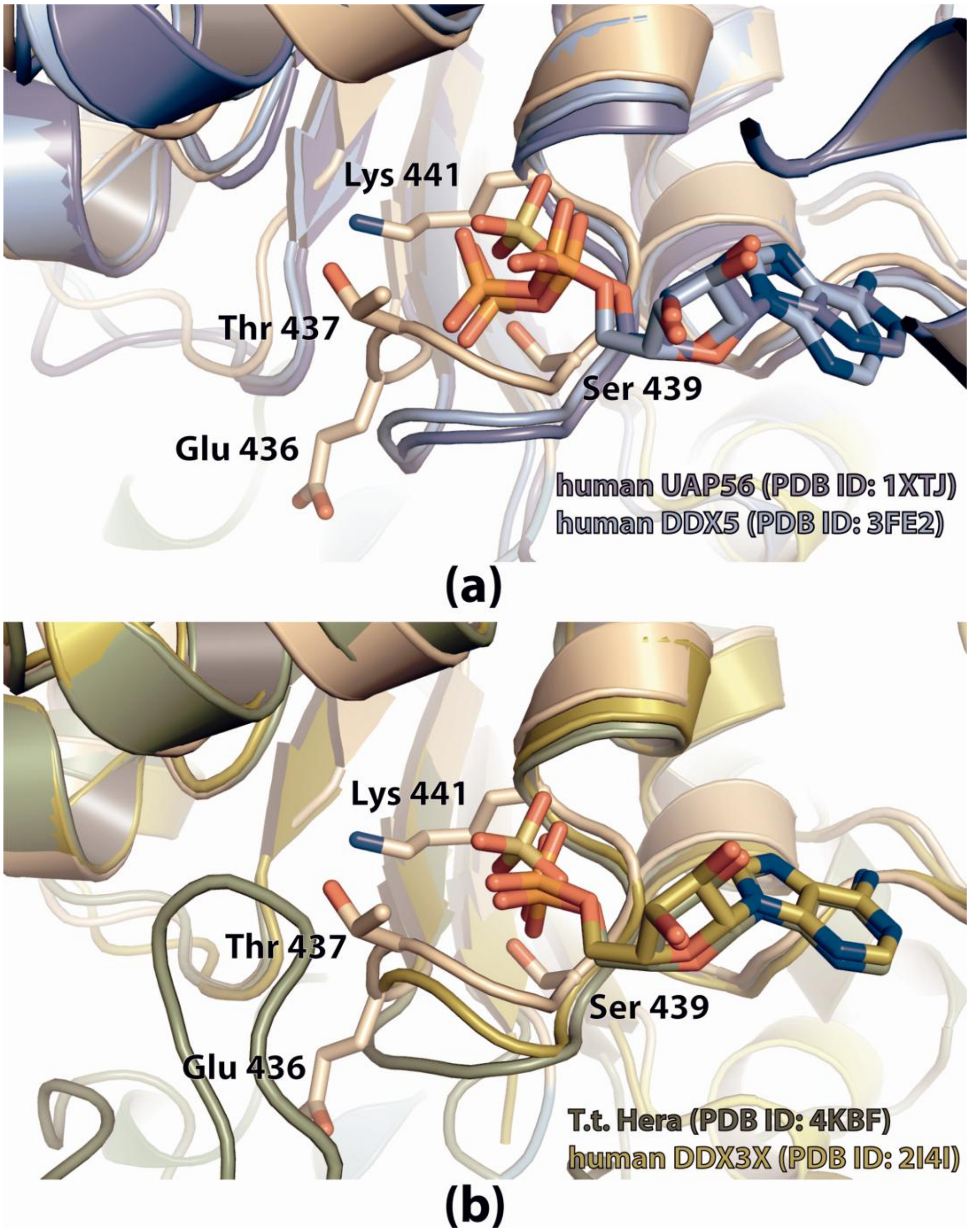

Figure S4 Different P-loop conformations observed for DEAD-box helicases with bound AMP and ADP molecules. (a) Superposition of hPrp28ΔN structure (wheat) with human UAP56 (PDB id: 1XTJ) and human DDX5 (PDB id: 3FE2). (b) Superposition of hPrp28ΔN structure (wheat) with *Thermus Thermophilus* helicase HERA (PDB id: 4KBF) and human DD3X (PDB id: 2I4I) colored olive. Sulfur ion, AMP and ADP molecules as well as side chain atoms of 3 residues constituting the P-loop in hPrp28 are depicted as sticks.
